# Women with type 1 diabetes gain more weight during pregnancy compared to age-matched healthy women despite a healthier diet: a prospective case–control observational study

**DOI:** 10.1007/s42000-023-00454-6

**Published:** 2023-05-26

**Authors:** Giuseppe Defeudis, Rossella Mazzilli, Domenico Benvenuto, Massimo Ciccozzi, Alfonso Maria Di Tommaso, Antongiulio Faggiano, Dario Tuccinardi, Mikiko Watanabe, Silvia Manfrini, Yeganeh Manon Khazrai

**Affiliations:** 1 Research Unit of Endocrinology and Diabetes, Università Campus Bio-Medico Di Roma, Rome, Italy; 2 Unit of Endocrinology, Department of Clinical and Molecular Medicine, Sapienza University of Rome, 00185 Rome, Italy; 3 Unit of Medical Statistics and Molecular Epidemiology, Università Campus Bio-Medico Di Roma, Rome, Italy; 4 Department of Experimental Medicine, Section of Medical Pathophysiology, Food Science and Endocrinology, Sapienza University of Rome, Rome, Italy; 5 Human Nutrition and Food Sciences, Università Campus Bio-Medico Di Roma, Rome, Italy

**Keywords:** Diabetes, **T**ype 1 diabetes mellitus, HbA1c, Pregnancy, Macrosomia, Nutrition

## Abstract

**Purpose:**

Women with type 1 diabetes mellitus (T1D), especially those with suboptimal glucose control, have 3–4 greater chances of having babies with birth defects compared to healthy women. We aimed to evaluate glucose control and insulin regimen modifications during the pregnancy of women with T1D, comparing the offspring’s weight and the mother’s weight change and diet with those of non-diabetic, normal-weight pregnant women.

**Methods:**

Women with T1D and age-matched healthy women controls (CTR) were consecutively enrolled among pregnant women with normal weight visiting our center. All patients underwent physical examination and diabetes and nutritional counseling, and completed lifestyle and food intake questionnaires.

**Results:**

A total of 44 women with T1D and 34 healthy controls were enrolled. Women with T1D increased their insulin regimen during pregnancy, going from baseline 0.9 ± 0.3 IU/kg to 1.1 ± 0.4 IU/kg (*p* = 0.009), with a concomitant significant reduction in HbA1c (*p* = 0.009). Over 50% of T1D women were on a diet compared to < 20% of healthy women (*p* < 0.001). Women with T1D reported higher consumption of complex carbohydrates, milk, dairy foods, eggs, fruits, and vegetables, while 20% of healthy women never or rarely consumed them. Despite a better diet, women with T1D gained more weight (*p* = 0.044) and gave birth to babies with higher mean birth weight (*p* = 0.043), likely due to the daily increase in insulin regimen.

**Conclusion:**

A balance between achieving metabolic control and avoiding weight gain is crucial in the management of pregnant women with T1D, who should be encouraged to further improve lifestyle and eating habits with the aim of limiting upward insulin titration adjustments to a minimum.

## Introduction

Gestational diabetes is associated with numerous adverse pregnancy outcomes [1], and glycemic control is needed to prevent these conditions [2]; in fact, poor glycemic control during pregnancy is associated with an increased risk of congenital abnormalities [3]. In particular, women with type 1 diabetes mellitus (T1D) have three to four times greater chances of giving birth to infants with severe birth defects compared to healthy women, including cardiac malfunction, neural tube and kidney defects, and gastrointestinal or limb abnormalities [4]. Farrell et al. [5] showed that higher glycated hemoglobin (HbA1c) is a significant prognostic factor as it increases the risk of malformations. Indeed, congenital abnormalities can occur in fetuses that have been exposed to hyperglycemia mainly in the first trimester, before organogenesis. Achieving good glycemic control through diet and insulin therapy is therefore crucial in pregnant women with T1DM [6, 7]. The fundamental objective outlined by the American Diabetes Association (ADA) is to achieve and maintain good glucose control through a healthy diet and regular exercise along with careful titration of insulin regimen based on frequent monitoring of glucose levels [8]. However, T1DM outpatient clinics may fall short in devoting enough time and effort to lifestyle counseling on one hand, and pregnant women may, on the other hand, not be receptive to improving their diet and physical activity to keep insulin up-titration to a bare minimum. For these reasons, glucose control can be difficult to achieve, while significant insulin titration may lead to unintended weight gain.

Therefore, in this real-life study based in Italy, as the primary outcome we aimed to explore the effectiveness on weight gain of routine counseling and management of pregnant women with T1D, comparing their nutritional habits with those of healthy pregnant women; the secondary outcome was evaluation of the impact of habitual routine management of blood glucose throughout pregnancy.

## Materials and methods

### Study design and population

This was a single-center, case–control, prospective observational study. Women with T1D aged 18–50 years were enrolled, while age-matched healthy women (CTR) were also consecutively enrolled among healthy pregnant women with normal weight visiting our center. All patients were prospectively followed up throughout pregnancy, underwent physical examination, received diabetes and nutritional counseling, and were asked to complete questionnaires on lifestyle (alcohol consumption, smoking, sleep, physical activity, water consumption, and HLA haplotype) and habitual food intake (food quality and frequency of consumption). Weight change was monitored and HbA1c was assessed by capillary electrophoresis at each follow-up visit. All women delivered at term between 38 and 41 weeks of gestation. The study protocol was approved by the local Ethics Committee (rif. 39.22) and conducted in accordance with the Declaration of Helsinki and good clinical practice (GCP). Written informed consent was obtained from the participants prior to enrolment.

### Statistical analysis

Statistical analysis was performed using version 7.0 of Graphpad (Graphpad Software, San Diego, CA, USA). Data are presented as mean ± SD for quantitative variables and as numbers and percentages for categorical variables. The Student’s t-test was used to compare continuous variables, and analysis of variance (ANOVA) with Bonferroni post-hoc test was performed when appropriate. Nominal variables were analyzed with the chi-square non-parametric test. *p* < 0.05 was considered statistically significant.

We calculated that with two groups of 25, assuming that the average weight gain in T1D patients is 9.6 ± 4.9 kg compared to the mean of 13 kg of the general population, the power would be 80% using an alpha cut-off value of 0.05 to detect a difference in weight between T1D and non T1D pregnant women. To allow for the possibility of subject dropout, we then recruited at least five additional subjects per group to have 30 eligible subjects per group contributing data.

## Results

Forty-four women with T1D and 32 healthy controls were enrolled. Age was similar in the two groups [36.4 (3.7) years vs 37.7 (4.9) years in the T1D and the CTR group, respectively; *p* = 0.121], as was mean body weight before pregnancy [59.5 (9.6) kg vs 58.3 (10.7) kg in the T1D and the CTR group, respectively; *p* = 0.662.].

Before pregnancy, women with T1D had a mean HbA1c (SD) of 6.7% (1.5) and were on a mean daily insulin regimen of 0.9 (0.3) IU/kg. In total, 38 out of 44 (86.4%) women were on a multiple daily injection regimen (MDI), whereas six out of 44 (13.6%) were on continuous subcutaneous insulin infusion (CSII). All control women had normal glucose tolerance (Table 1). The daily insulin regimen of women with T1D increased throughout pregnancy (1.1 ± 0.4 IU/kg vs. 0.9 ± 0.3 IU/kg, *p* = 0.009). Insulin was titrated upwards in 38/44 (86.4%) women, while in 4/44 (9.1%) the schedule remained unchanged and only 2/43 (4.5%) had it reduced. A significant reduction in HbA1c was observed by the end of pregnancy [6.7 (1.5) % vs. 6.0 (0.9) %, *p* = 0.009)]. Women with T1D gained more weight compared to healthy women throughout pregnancy (13.2 ± 4.4 kg vs. 11.4 ± 4.3 kg, *p* = 0.044), with a higher mean birth weight in T1D mothers’ offspring compared to healthy mothers’ babies (3.5 ± 0.4 kg vs 3.3 ± 0.4 kg, *p* = 0.043). A non-significant (but suggestive) correlation was found between daily insulin regimen and weight gain (r = 0.32, *p* = 0.123).

**Table 1:** Clinical data of the study groups type 1 diabetes (T1D) patients and healthy controls (CTR). Data were analyzed by Student’s t-test for continuous variables and by the chi-square test for categorical variables

	T1D	CTR	*p*
	*N. 44*	*N. 32*	
Age, years (mean ± SD)	36.4 ± 3.7	37.7 ± 4.9	*0.121*
Body weight, Kg (mean ± SD)—*before pregnancy*	59.5 ± 9.6	58.3 ± 10.7	*0.662*
Weight gain, Kg (mean ± SD)	13.2 ± 4.4	11.4 ± 4.3	*0.044*
Newborn body weight, Kg (mean ± SD)	3.5 ± 0.4	3.3 ± 0.4	*0.043*
Alcohol consumption, N. (%)—*before pregnancy*	3 (6.8)	5 (15.6)	*0.363*
Smoking, N. (%)—*before pregnancy*	7 (15.9)	6 (18.8)	*0.613*
Night resting, hours (mean ± SD)—*before pregnancy*	7.4 ± 1.5	7.4 ± 1.3	*0.990*
Physical activity, N. (%)			
* before pregnancy*	11 (25)	10 (31.3)	*0.421*
* during pregnancy*	3 (6.8)	4 (12.5)	*0.253*
* light (*< *3 times/week)*	31 (70.5)	19 (59.4)	*0.334*
* moderate (at least 3 times/week)*	13 (29.5)	13 (40.6)	*0.152*
Eating Habits, N. (%)- *before pregnancy*			
* On a prescriptive diet*	24 (54.5)	5 (15.6)	< *0.001*
* Eating out*	12 (27.3)	9 (28.1)	*0.891*
* Snacks*	18 (40.9)	13 (40.6)	*0.992*
Water consumption, N. (%)—*before pregnancy*			
< *1 l / day*	6 (13.6)	3 (9.4)	*0.301*
> *1 l / day*	38 (86.4)	29 (90.6)	*0.702*

There were no significant differences between the two groups in relation to lifestyle, smoking habits, alcohol consumption, HLA + , HLA –, and sleep time (Tables 1 and 2**).** Indeed, over half of the women with T1D were on an individualized dietary regime compared to less than 20% of healthy women (*p* < 0.001).

**Table 2:** Food frequency questionnaire in type 1 diabetes mellitus (T1D – N. 44) patients and healthy controls (CTR – N. 32). Data were analyzed with the chi-square test

	*Never / Rarely* *(%)*	*At least 3 times/week* *(%)*	*Daily* *(%)*
Complex carbohydrates^a^			
* T1D*	2	11	86
* CTR*	3	25	72
Simple sugars			
* T1D*	64	20	16
* CTR*	56	25	19
Fruits and vegetables^b^			
* T1D*	0	0	100
* CTR*	3	9	88
Meat			
* T1D*	2	45	52
* CTR*	3	56	41
Dairy foods^c^			
* T1D*	11	7	82
* CTR*	0	9	91
Fish			
* T1D*	20	75	5
* CTR*	19	81	0
Eggs^d^			
* T1D*	9	89	2
* CTR*	28	72	0

^a^*p* = 0.013; ^b^*p* < 0.001; ^c^*p* = 0.043; ^d^*p* < 0.001

**Fig. 1:**
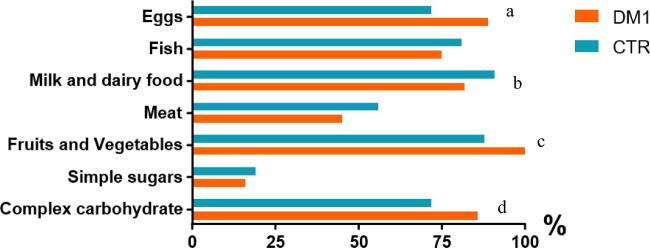
Differences between T1D and CTR in terms of daily consumption of carbohydrates (complex and simple), fruit/vegetables, and milk/derivates and weekly consumption of meat, fish, and eggs have been evaluated using the chi-square test. ^a^*p* < 0.001; ^b^*p* = 0.043; ^c^*p* < 0.001; ^d^*p* = 0.013

No significant differences were found in the consumption of sugar-rich foods, meat, and fish (Table 2). Women with T1D reported higher daily consumption of simple and complex carbohydrates (*p* = 0.013), fruits and vegetables (*p* < 0.001), dairy (*p* = 0.043), and eggs (*p* < 0.001) (Table 2) (Fig. 1**)**. Interestingly, 20% of healthy women never or rarely consumed such foods during their pregnancy.

## Discussion

It is common knowledge that pregnancy is a difficult time in which to achieve good glucose control because of placental hormones, growth factors, and cytokine production together with an increase in insulin resistance [9]. Even women with good dietary knowledge often find glucose control harder to achieve, especially during the early stages of pregnancy when they might be suffering from morning sickness, nausea, or vomiting. Studies have shown that daily insulin requirements may decrease during the first three months of pregnancy as the fetus uses the mother’s body glucose [10]. Hypoglycemia frequently occurs at this stage and up to 70% of women with T1D report such episodes during pregnancy [11]. The high rate of macrosomic babies notwithstanding tight glycemic control has also been attributed to frequent rebound hyperglycemic episodes following hypoglycemia as well as to the hyperglycemia itself [12]. It is therefore advisable for diabetologists to closely follow their patients during pregnancy [13], adequately informing them about the possibility of rapid fluctuations in insulin requirements [14]. To prevent hyper- or hypoglycemia, dietary and lifestyle counseling is crucial, although insulin regimen titration is often the mainstay of treatment. We herein show that even though over half of the women with T1D had been on an individualized diet since before pregnancy, had received adequate counseling on the importance of controlling rapid weight gain to avoid hyperglycaemia and macrosomic babies, and had followed a reported reasonably good dietary regime throughout pregnancy, insulin up-titration was required to maintain good glucose control so as to prevent significant weight gain. Moreover, newborns of women with T1D still had significantly higher body weight compared to controls. This could be due to weight gain or rebound hyperglycemia or is possibly attributable to the increased insulin dosage per se [15]. On the other hand, weight gain could occur for other reasons apart from insulin effects on adipose tissue, including prepregancy lifestyle, smoking habits, alcohol consumption, and sleep time, and this could be a confounder. In this study, none of these aspects appears to be related to weight.

Several other factors have been proposed as being associated with higher gestational weight gain, such as unfavorable obstetric history, prepregnancy overweight/obesity, gestational age at delivery, and length of follow-up [16]. On the other hand, appetite during pregnancy is increased and feedback responses to metabolic hormones, including both leptin and insulin, are suppressed to promote a positive energy balance [17]. Furthermore, hyperemesis, which frequently occurs in pregnant women [18], induces increased consumption of carbohydrates.

All things considered, it seems reasonable to recommend tailored and careful management of pregnant women with normal weight and T1D in order to keep insulin up-titration to a bare minimum. However, a weak correlation was found between daily insulin regimen and weight gain. This should be pursued by further improving nd diet and regular physical activity, with body weight gain being taken into consideration together with glucose control per se, although further, larger studies are warranted to deepen our understanding of the underlying mechanisms regarding this aspect.

In our cohort, women with T1D exhibited a 0.7% mean decrease in HbA1c throughout pregnancy. It is noteworthy that women without diabetes show a reduction in HbA1c levels during the first trimester as a decrease in red blood cell life span. Similarly, women with T1D seem to experience a slight reduction independent of glucose control due to the same mechanism [19]. Our results are therefore likely due to the increased insulin dosage on top of the aforementioned impact of red blood cell life span. However, more studies are needed to investigate HbA1c reduction during pregnancy and its correlation with glycemic control.

Our study has some limitations. First, a relatively small cohort was enrolled, although it exceeded the sample size calculated a priori. Moreover, several parameters were not recorded, such as the duration of diabetes, lipid profile, and/or continuous glucose monitoring data, which set a limit on the in-depth evaluation of a number of factors. In addition, height was not recorded in several women, making impossible the calculation of BMI for the entire cohort. However, one of the inclusion criteria for this study was normal weight, and, therefore, women with overweight, obesity, or underweight were not initially enrolled. Furthermore, physical activity and dietary habits were not directly monitored and only self-reported, possibly creating bias. In addition, insulin resistance could equally occur during pregnancy in healthy subjects; in this regard, comparing patients with T1DM to women without gestational diabetes is not optimal. However, it was our decision to compare our study group with a healthy control group and to exclude women with gestational diabetes, since, in this case, the diagnosis occurs later (at least at 16–18 weeks of gestation or even at 24–28 weeks of gestation) and, therefore, we could not compare the baseline characteristics having different times of diagnosis. Furthermore, the mechanisms by which variations in body weight could exist are very different among women with and without T1DM and the therapeutic approach would vary widely. Finally, the time in range, which is now considered an important parameter for monitoring diabetes in pregnancy, was not recorded.

Our study also features some strengths. As a single-center study, all women underwent the same counseling given by the same health professionals, significantly limiting heterogeneity both in the assessment and in the management of the patients. Moreover, the population was relatively homogeneous in terms of ethnicity, age, education, and habits, contributing to a more solid interpretation of the results.

Pregnancy care in women with diabetes is very complex and should be dealt with by a multidisciplinary team involving diabetologists, gynecologists, and obstetricians, as well as registered dietitians. It is, moreover, crucial to provide mothers with the skills to cope with issues concerning their pregnancy, as their education can play a significant role in improving pregnancy outcomes. To address the issue by simply increasing insulin doses is to ? grossly underestimate the problem, while more attention should be paid to diet and physical activity in the effort to achieve body weight control in the management of pregnant women with T1D.

Pregnancy care in women with diabetes is extremely complex. To address the issue by simply increasing insulin doses is to grossly underestimate the problem. Instead, it should be dealt with by a multidisciplinary team involving diabetologists, gynecologists, and obstetricians, as well as registered dietitians. It is, moreover, crucial to provide mothers with the skills to cope with issues concerning their pregnancy, as their education can play a significant role in improving pregnancy outcomes. Finally, pregnant women with T1D must be encouraged to pay close attention to diet and physical activity to enable them to achieve body weight control during this critical period.

## Data Availability

Data will be made available upon reasonable request to the corresponding author.
